# Cyclooxygenase-2 induced β1-integrin expression in NSCLC and promoted cell invasion via the EP1/MAPK/E2F-1/FoxC2 signal pathway

**DOI:** 10.1038/srep33823

**Published:** 2016-09-22

**Authors:** Jinshun Pan, Qinyi Yang, Jiaofang Shao, Li Zhang, Juan Ma, Yipin Wang, Bing-Hua Jiang, Jing Leng, Xiaoming Bai

**Affiliations:** 1 Department of Biotherapy, The Second Affiliated Hospital, Nanjing Medical University, Nanjing, Jiangsu 210011, P.R. China; 2 Cancer Center, Department of Pathology, Nanjing Medical University, Nanjing 210029, P.R. China; 3 Department of Pathology, The First Affiliated Hospital, Nanjing Medical University, Nanjing 210029, P.R. China; 4 Department of Bioinformatics, Nanjing Medical University, Nanjing 210029, P.R. China; 5 Department of Pathology, Anatomy and Cell Biology, Thomas Jefferson University, Philadelphia, Pennsylvania PA 19107, USA

## Abstract

Cyclooxygenase-2 (COX-2) has been implicated in cell invasion in non-small-cell lung cancer (NSCLC). However, the mechanism is unclear. The present study investigated the effect of COX-2 on β1-integrin expression and cell invasion in NSCLC. COX-2 and β1-integrin were co-expressed in NSCLC tissues. COX-2 overexpression or Prostaglandin E2 (PGE2) treatment increased β1-integrin expression in NSCLC cell lines. β1-integrin silencing suppressed COX-2-mediated tumour growth and cancer cell invasion *in vivo* and *in vitro*. Prostaglandin E Receptor EP1 transfection or treatment with EP1 agonist mimicked the effect of PGE_2_ treatment. EP1 siRNA blocked PGE_2_-mediated β1-integrin expression. EP1 agonist treatment promoted Erk1/2, p38 phosphorylation and E2F-1 expression. MEK1/2 and p38 inhibitors suppressed EP1-mediated β1-integrin expression. E2F-1 silencing suppressed EP1-mediated FoxC2 and β1-integrin upregulation. ChIP and Luciferase Reporter assays identified that EP1 agonist treatment induced E2F-1 binding to FoxC2 promotor directly and improved FoxC2 transcription. FoxC2 siRNA suppressed β1-integrin expression and EP1-mediated cell invasion. Immunohistochemistry showed E2F-1, FoxC2, and EP1R were all highly expressed in the NSCLC cases. This study suggested that COX-2 upregulates β1-integrin expression and cell invasion in NSCLC by activating the MAPK/E2F-1 signalling pathway. Targeting the COX-2/EP1/PKC/MAPK/E2F-1/FoxC2/β1-integrin pathway might represent a new therapeutic strategy for the prevention and treatment of this cancer.

Lung cancer is the most common cancer in terms of incidence and death rate worldwide^1^. The dismal five-year survival rate is 10–15% worldwide and 14–17% in the United States, which has shown little improvement over the past three decades^1^,^2^,^3^,^4^. Although a combination of resection and chemotherapy can improve survival, the prognosis of lung caner is still extremely poor, and often associated with malignant invasion and metastasis^1^,^5^.

Cyclooxygenase-2 (COX-2) has been proposed as an important cellular factor associated with carcinogenesis and progression in many types of cancers. Previous studies indicated that COX-2 expression was upregulated in many cancer tissues^6^,^7^,^8^ and that Prostaglandin E2 (PGE2), one of most important products of COX-2, increased cancer cell growth, migration and invasion^8^,^9^,^10^. In non-small-cell lung cancer (NSCLC), higher levels of COX-2 expression are found at most stages of tumour progression compared with normal lung tissue, which is considered a negative predictor of survival^11^,^12^,^13^. Increased COX-2 expression is frequently seen in precursor lesions of human adenocarcinoma of the lung, which might be associated with an invasive and more aggressive phenotype of lung cancer^12^. New targets aimed at cellular COX-2/PGE2 signalling pathways have provided therapeutic strategies for the treatment of NSCLC metastasis^12^,^13^.

Integrins are a family of transmembrane cellular receptors that mediate cell–cell and cell–matrix interactions. They are heterodimeric glycoproteins that serve as adhesion receptors for extracellular matrix proteins and also transduce biochemical signals into the cell. These receptors comprise an α and a β subunit^14^. Integrins of the β1-family mainly transduce signals from the extracellular matrix to modulate growth and differentiation^14^,^15^, and has been implicated in cell proliferation, adhesion and metastasis in a wide variety of human cancers, including breast, colon and ovary^16^,^17^.

To date, little has been discovered about the association between COX-2 and β1-integrin expression in lung cancer. Therefore, we aimed to investigate the effect of COX-2 on β1-integrin expression and cell invasion in NSCLC. The current study suggested that COX-2 upregulates β1-integrin expression and cell invasion in NSCLC cells through the Prostaglandin E Receptor EP1, and the PKC/MAPK/E2F-1/FoxC2 signalling pathway might be involved in COX-2-mediated β1-integrin upregulation.

## Results

### COX-2 promotes β1-integrin expression and cell invasion in NSCLC cells

**Figure 1:**
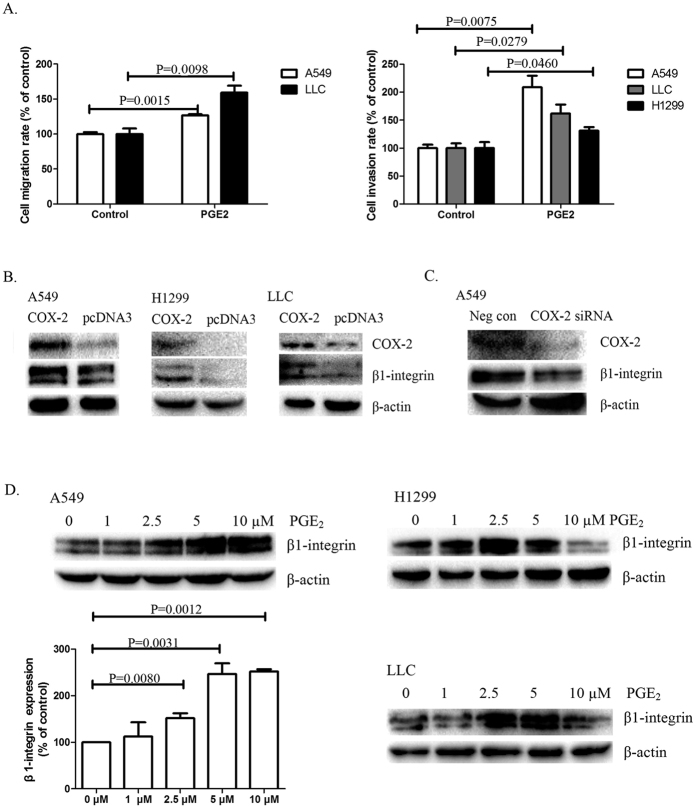
COX-2 promotes β1-integrin expression in NSCLC cells. (**A**) Effects of PGE2 on cell migration and invasion in NSCLC cells. The cell migration and invasion assays were performed in 12-well transwell units. A549, LLC and H1299 cells were treated with 5 μM PGE2. Results are presented as the mean ± SEM (n = 3). (**B**) Effects of COX-2 overexpression on β1-integrin regulation in NSCLC cells. A549, H1299 and LLC cells were transfected with COX2-pcDNA3 plasmid or empty pcDNA3 plasmid. Total protein was isolated and visualized using anti-β1-integrin and anti-COX-2 antibodies. Levels of β-actin served as a loading control. (**C**) Effects of COX-2 RNAi on β1-integrin regulation in NSCLC cells. A549 cells were transfected with an EP1R-siRNA. After 72 h, total protein was isolated and visualized using anti-β1-integrin and anti-COX-2 antibodies. Levels of β-actin served as a loading control. (**D**) Effects of PGE2 treatment on β1-integrin expression in NSCLC cells. A549 cells were treated with PGE2 at various concentrations for 24 h. Total protein was isolated and visualized using anti-β1-integrin antibody. Levels of β-actin served as a loading control. Results are presented as the mean ± SEM (n = 3). Similar effects were shown in LLC and H1299 cells. These experiments were performed three times with similar results.

Three lung cancer cell lines, A549, H1299, and LLC were treated with PGE2. Among them, A549 and H1299 are human lung cancer cell lines, and LLC is a mouse lung cancer cell line. The migration and invasion assays showed that PGE2 promoted cell migration and invasion in these NSCLC cell lines (Fig. 1A). The effects of PGE2 treatment on cell viability were shown in Supplement Figure S1.

To detect if COX-2 overexpression induce β1-integrin upregulation in NSCLC cells, A549, H1299, and LLC cells were transfected with the COX2-pcDNA3 plasmid. Figure 1B shows that β1-integrin expression was significantly upregulated in the cells stably expressing COX-2 (compared with the control cells). By contrast, COX-2 siRNA decreased β1-integrin expression (Fig. 1C). Exogenous PGE2 treatment significantly increased β1-integrin expression in the three NSCLC cell lines (Fig. 1D).

### β1-integrin expression is involved in COX-2-induced cell invasion in NSCLC cells

**Figure 2:**
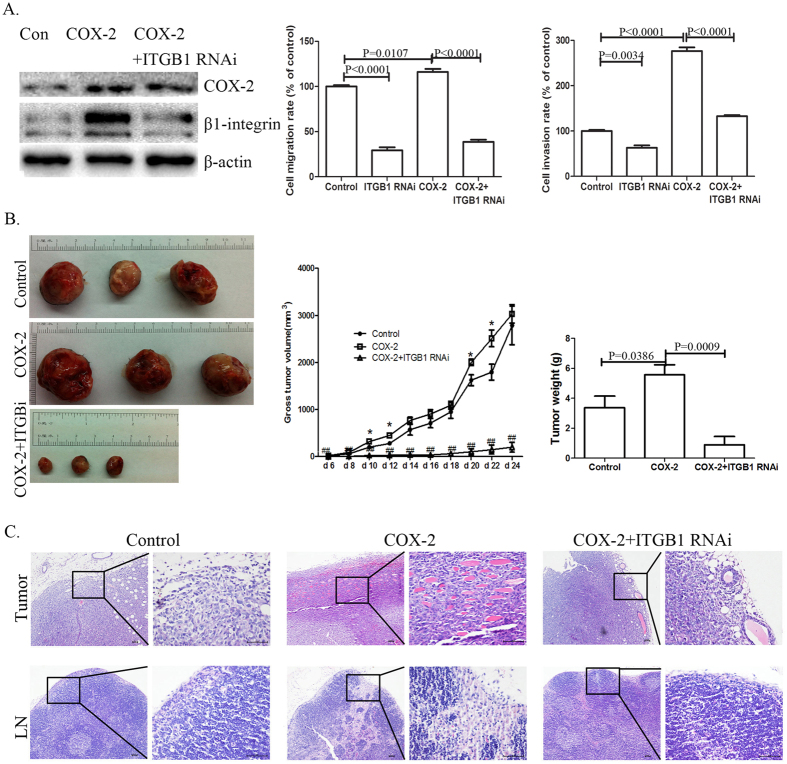
β1-integrin is involved in COX-2-induced tumour growth and cell invasion in NSCLC. (**A**) Effects of β1-integrin expression on COX-2-induced cell migration and invasion in LLC cells. COX-2-transfected LLC cells were infected with lentivirus silencing the β1-integrin expression (termed COX-2 + ITGB1 RNAi cells). Total protein was isolated and visualized using anti-β1-integrin and anti-COX-2 antibodies. Levels of β-actin served as a loading control. The cell migration and invasion assays were performed in LLC control cells, ITGB1 RNAi cells, COX-2 overexpression cells and COX-2 + ITGB1 RNAi cells. Results are presented as the mean ± SEM (n = 3). (**B**) Effects of β1-integrin expression on COX-2-induced tumour growth and cell invasion in xenograft models. Four-week-old female C57/B6 mice were randomized into three groups (n = 8). Group 1 was implanted with wild-type LLC cells (control group). Group 2 was implanted with COX-2 overexpression cells. Group 3 was implanted with COX-2 + ITGB1 RNAi cells. Representative images of tumours from each group were shown. The tumour growth curve and a comparison of average tumour weight on the final day among three groups are shown. (**C**) HE staining of tumours and adjacent lymph nodes (LN) sections showing histopathological features of the xenografts in mice. Scale bars = 100 μm (10×). Scale bars = 50 μm (40×).

We then infected COX-2 transfected LLC cells with a lentivirus construct to silence β1-integrin expression. β1-integrin RNA interference diminished cell migration and invasion *in vitro*; COX-2 overexpression improved cell migration and invasion. Silencing of β1-integrin expression blocked COX-2-promoted migration and invasion in LLC cells (Fig. 2A).

Following the establishment of these cell lines, we established tumour xenografts by subcutaneously implanting these LLC cell lines into normal C57/B6 mice. Analysis of implanted tumour xenografts revealed that COX-2 overexpression not only induced tumour growth, but also promoted cancer cell invasion into adjacent muscular tissues. Furthermore, COX-2 overexpression in implanted tumour xenografts induced proliferation of adjacent lymphatic network. However, β1-integrin downregulation significantly reduced COX-2-mediated tumour growth and invasion (Fig. 2B,C).

### Positive correlation between COX-2 and β1-integrin in lung cancer tissues

**Figure 3:**
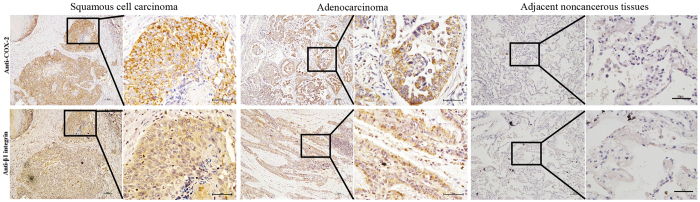
The expression of COX-2 and β1-integrin in NSCLC cancer tissues and adjacent noncancerous tissues. Representative immunohistochemical images of lung squamous cell carcinoma tissues, adenocarcinoma tissues and adjacent noncancerous tissues stained with the anti-human COX-2 and β1-integrin antibodies. Scale bars = 100 μm (10×). Scale bars = 50 μm (40×). The integrated grey level was carried out using Q-Win software. Statistical analysis of integrated grey levels of 80 samples was performed using STATA se12.0 software. β1-integrin and COX-2 comparisons between NSCLC and control tissue groups were analysed by Shapiro–Wilk’s W test. Spearman’s correlation assay showed that β1-integrin and COX-2 expression displayed significant positive correlations, both in the squamous cell carcinoma samples (Spearman’s rho = 0.789, *P* < 0.01) and in the adenocarcinoma samples (Spearman’s rho = 0.634, *P* < 0.01).

Using immunohistochemistry, 32 cases (80%) showed positive COX-2 and 34 cases (85%) showed positive β1-integrin expression in the cytoplasm in adenocarcinoma tissues. 34 cases (85%) showed positive COX-2 and 35 cases (87.5%) showed positive β1-integrin expression in squamous cell carcinoma tissues. A strong positive correlation existed between the expression levels of β1-integrin and COX-2 protein in adenocarcinoma and squamous cell carcinoma tissues (Fig. 3). Negative COX-2 and β1-integrin expression were detected in tissues adjacent to the cancer. β1-integrin and COX-2 comparisons between squamous cell carcinoma and adenocarcinoma groups were analysed by Shapiro–Wilk’s W test. The data distribution was not normal. Spearman’s correlation assay showed that the data were statistically significant. The clinicopathological features of the NSCLC patients are listed in Tables 1 and 2. The levels of COX-2 and β1-integrin expression correlated significantly with the lung cancer stages. The level of COX-2 expression correlated significantly with lymph nodes metastasis in adenocarcinoma tissues.

### The EP1 receptor is involved in COX-2-mediated β1-integrin expression and cell migration in NSCLC cells

**Figure 4:**
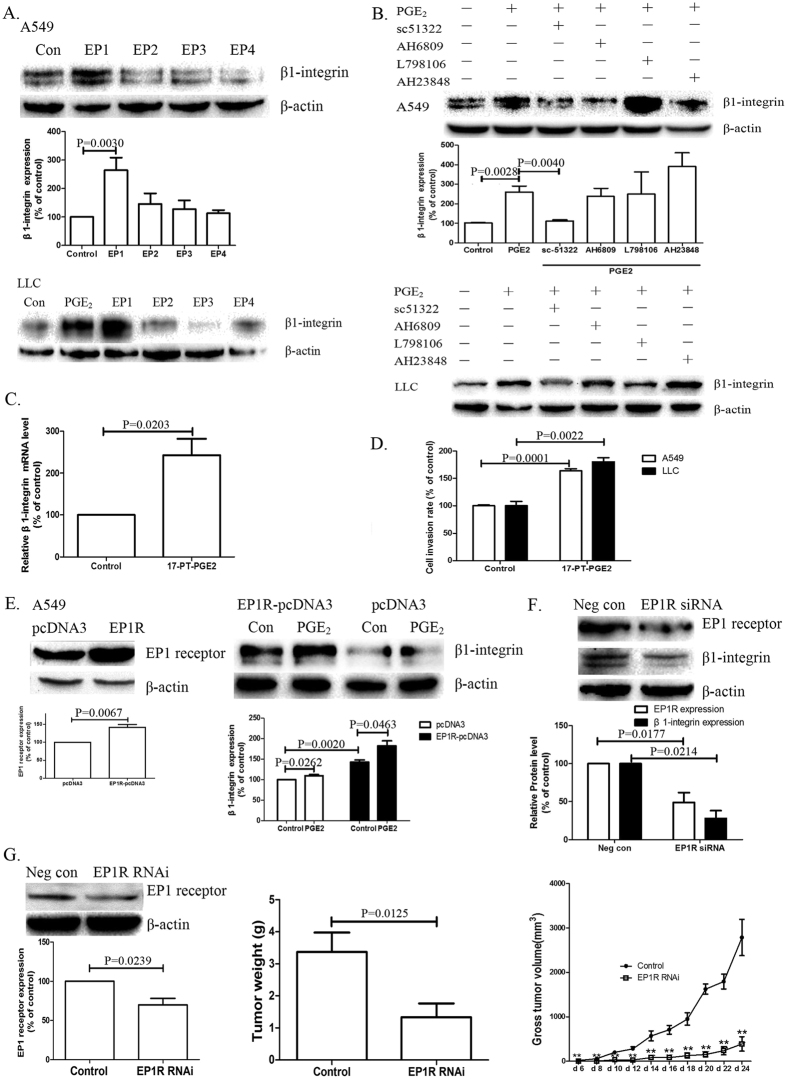
EP1 receptor is involved in COX-2-induced β1-integrin expression in NSCLC. (**A**) Effects of EP agonists on β1-integrin expression in NSCLC cells. A549 cells were exposed to 5 μM agonists for EP1 (17-PT-PGE2), EP2 (butaprost), EP3 (sulprostone) and EP4 (PGE1 alcohol) for 24 h, respectively. (**B**) Effects of EP antagonists on PGE2-mediated β1-integrin expression in NSCLC cells. A549 cells were pre-treated with various EP antagonists for 1 h, followed by PGE2 for 24 h (antagonists for EP1 (sc15322), EP2 (AH6809), EP3 (L-798106) and EP4 (AH23848)). Similar effects were observed in LLC cells. (**C**) The effect of EP1 agonist on β1-integrin transcription in A549 cells. Total RNA was isolated, and real-time PCR analysis was performed. GAPDH served as a loading control. Results are shown as the mean ± SEM (n = 3). (**D**) Effects of EP1 agonist on NSCLC cell invasion. Results are presented as the mean ± SEM (n = 3). (**E**) Effects of EP1R overexpression on β1-integrin regulation in NSCLC cells. EP1R-transfected A549 cells were exposed to PGE2 for 24 h. Results are presented as the mean ± SEM (n = 3). (**F**) Effects of EP1R RNAi on β1-integrin regulation in NSCLC cells. A549 cells were transfected with an EP1R-siRNA. Results are presented as the mean ± SEM (n = 3). (**G**) Effects of EP1R expression on tumour growth in xenograft models. LLC cells were infected with lentivirus silencing EP1R expression (termed EP1R RNAi cells). Four-week-old female C57/B6 mice were randomized into two groups (n = 8). Group 1 was implanted with wild-type LLC cells (control group). Group 2 was implanted with EP1R RNAi cells. Representative images of tumours from each group, the tumour growth curve and a comparison of average tumour weight on the final day between two groups are shown.

A549 and LLC cells were treated with EP1, EP2, EP3 and EP4 receptor agonists. Figure 4A shows that treatment with butaprost (EP2 agonist), sulprostone (EP3 agonist) and PGE1 alcohol (EP4 agonist), respectively, had little or no effect on β1-integrin expression. By contrast, treatment with 17-PT-PGE2, a specific agonist of the EP1 receptor, significantly enhanced β1-integrin expression. Pretreatment with antagonists of EP receptors in A549 or LLC cells showed little effect on PGE2-mediated β1-integrin upregulation, except for treatment with sc-51322, a specific antagonist of the EP1 receptor, which markedly blocked PGE2-mediated β1-integrin upregulation (Fig. 4B). Figure 4C showes that 17-PT-PGE2 treatment increased β1-integrin mRNA expression by over 2-fold.

At the same time, 17-PT-PGE2 promoted cell invasion effectively in various NSCLC cell lines (Fig. 4D). To corroborate the role of the EP1 receptor (EP1R) in the induction of β1-integrin expression, A549 cells were transfected with the EP1R-pcDNA3 construct. Figure 4E shows that EP1R overexpression increased the basal expression level of the β1-integrin protein. In addition, β1-integrin expression was significantly upregulated in the EP1R-transfected cells when treated with PGE2, compared with the control cells.

To further study the specific role of EP1R in β1-integrin expression, A549 cells and LLC cells were treated with RNA interference constructs targeting EP1R. As shown in Fig. 4F, siRNAs targeting EP1R greatly reduced the β1-integrin expression in A549 cells. We then infected LLC cells with lentivirus constructs that silenced EP1R expression. We then established tumour xenografts by implanting these LLC cell lines subcutaneously into normal C57/B6 mice. Analysis of the implanted tumour xenografts revealed that EP1R downregulation significantly reduced tumour growth; at the same time, no proliferation of adjacent lymphatic network was found in implanted tumour xenografts (Fig. 4G).

### New mechanism of β1-integrin expression regulated by E2F-1 and FoxC2 in NSCLC cells

**Figure 5:**
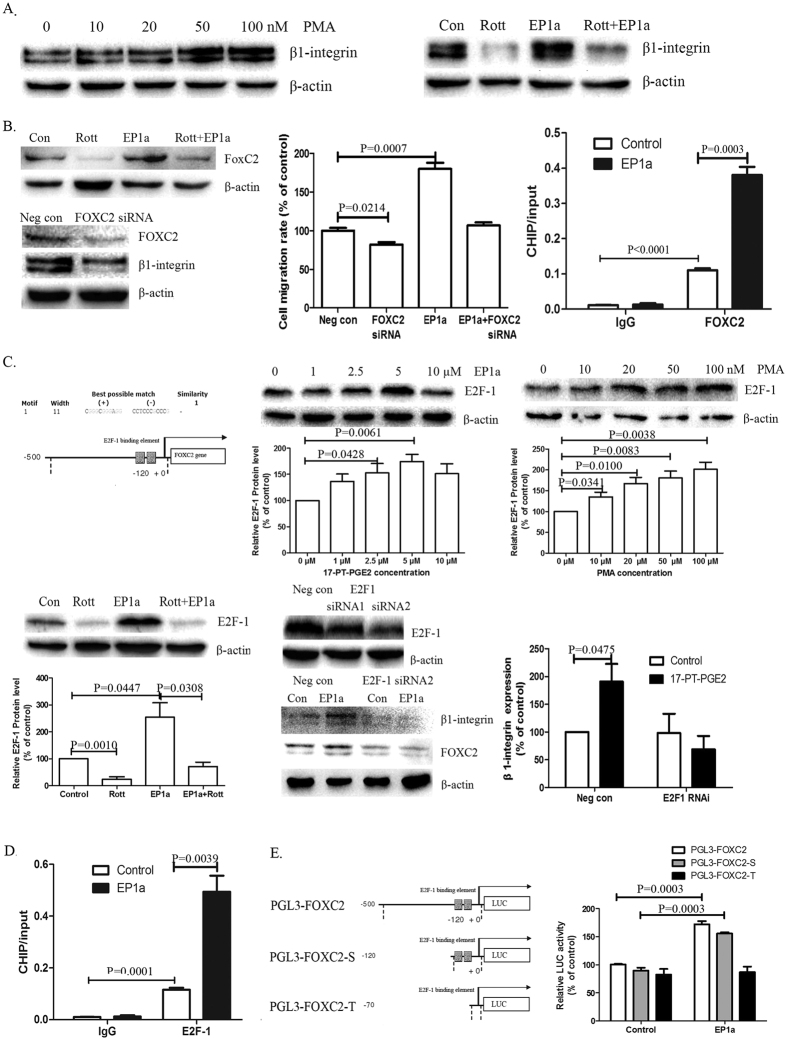
PKC/E2F-1/FoxC2 is involved in EP1R-induced β1-integrin expression in NSCLC. (**A**) Effects of PKC activation on β1-integrin expression in NSCLC cells. A549 cells were treated with PKC activator PMA for 24 h. A549 cells were pre-treated with PKC inhibitor Rottlerin for 1 h, followed by EP1 agonist (EP1a) for 24 h. These experiments were performed three times with similar results. (**B**) Effects of FoxC2 on EP1R-mediated β1-integrin expression in NSCLC cells. A549 cells were pre-treated with Rottlerin for 1 h, followed by EP1 agonist (EP1a) for 24 h. A549 cells were transfected with a FoxC2-siRNA, and then the cells were treated with 17-PT-PGE2 5 μM. The cell migration assays were performed in 12-well transwell units. Results are presented as the mean ± SEM (n = 3). ChIP assays were performed using anti-FoxC2 antibodies and anti-IgG, and followed by analyse of realtime-PCR. Results are presented as the mean ± SEM (n = 3). (**C**) The sequences of the human FoxC2 promoter were analysed in detail by the Motif Alignment & Search Tool. The putative E2F-1 binding element is located in the human FoxC2 promoter region. A549 cells were treated by EP1a or PMA at various concentrations. A549 cells were pre-treated with Rottlerin for 1 h, followed by EP1a for 24 h. Results are presented as the mean ± SEM (n = 3). A549 cells were transfected with E2F-1-siRNAs. A549 E2F-1-siRNA cells were exposed to EP1a for 24 h. Results are presented as the mean ± SEM (n = 3). (**D**) ChIP assays were performed using anti-E2F-1 antibodies and anti-IgG, and followed by analyse of realtime-PCR. Results are presented as the mean ± SEM (n = 3). (**E**) Effects of E2F-1 on FoxC2 transcription activity. Schematic diagram of luciferase reporter constructs for the FoxC2 promoter. A549 cells were transfected with wild-type or truncated FoxC2 promoter constructs, followed by EP1a treatment. Luciferase (Luc) activity was measured using the Promega Dual-Luciferase Reporter Assay system. Results are presented as the mean ± SEM (n = 3).

Our previous studies showed that EP1R activated the PKC signalling pathway in hepatocellular carcinoma cells^18^,^19^. In the current study, the PKC activator PMA significantly increased β1-integrin expression in A549 cells; whereas, the PKC inhibitor Rottlerin suppressed EP1-mediated β1-integrin expression significantly (Fig. 5A).

FoxC2, one of winged helix/forkhead transcription factors, is involved in PKC-mediated β1-integrin expression^18^. In the chromatin-immunoprecipitation (ChIP) assays, FoxC2 protein bound to an F-box element (FBE) in the integrin β1 promoter in a sequence-specific manner^20^. In the current study, FoxC2 siRNA suppressed β1-integrin expression and EP1R-mediated cell migration in A549 cells; the PKC inhibitor Rottlerin suppressed EP1R-mediated FoxC2 expression significantly (Fig. 5B). To determine if there is a direct interaction between FoxC2 and β1-integrin promoter, we analysed the sequences of human β1-integrin promoter in detail using Motif Alignment & Search Tool. ChIP-qPCR assay was performed. Treatment of A549 cells with EP1 agonist improved FoxC2 binding to the promoter of β1-integrin (Fig. 5B).

To identify the element that is involved directly in the induction of FoxC2 expression, we analysed the sequences of human FoxC2 promoter in detail using Motif Alignment & Search Tool. Interestingly, the FoxC2 promoter contains two putative E2F-1-binding elements at position −120 bp from the transcription initiation site of the FoxC2 gene, as shown in Fig. 5C. To investigate whether E2F-1 is involved in EP1-mediated β1-integrin expression, we detected the effects of EP1 activation on E2F-1 expression. Both the EP1 agonist and PMA improved E2F-1 expression significantly. Rottlerin suppressed EP1R-mediated E2F-1 expression significantly.

To further study the specific role of E2F-1 in β1-integrin expression, A549 cells were transfected with E2F-1 siRNAs. Depletion of the E2F-1 did not reduce the basal level of β1-integrin or the FoxC2 protein; however, EP1R-induced β1-integrin and FoxC2 expression were completely blocked in the E2F-1 siRNA-transfected cells.

ChIP-qPCR assay was performed to detect the direct binding between E2F-1 and FoxC2 promoter. As shown in Fig. 5D, treatment of A549 cells with EP1 agonist improved E2F-1 binding to the promoter of FoxC2. To clarify whether E2F-1 upregulates FoxC2 transcription by direct binding to specific elements in FoxC2 promoter, we generated a wild-type FoxC2 promoter construct (−500 to 0 bp) containing two putative E2F-1 elements (CCTCCCGCCCG) using the pGL3-basic vector (Fig. 5E). The luciferase reporter assay revealed that the activity of the wild-type FoxC2 promoter was significantly increased after EP1 agonist treatment in A549 cells. The FoxC2 promoter construct (−120 to 0 bp), containing two putative E2F-1 elements, was also generated in the pGL3-basic vector (short FoxC2 promoter-PGL3). It mimicked the effect of wild-type FoxC2 promoter after EP1 agonist treatment. Furthermore, we found that the truncated FoxC2 promoter (−70 to 0 bp, lacking the putative E2F-1 binding sites) showed no induction of luciferase activity under EP1 agonist treatment.

**Figure 6:**
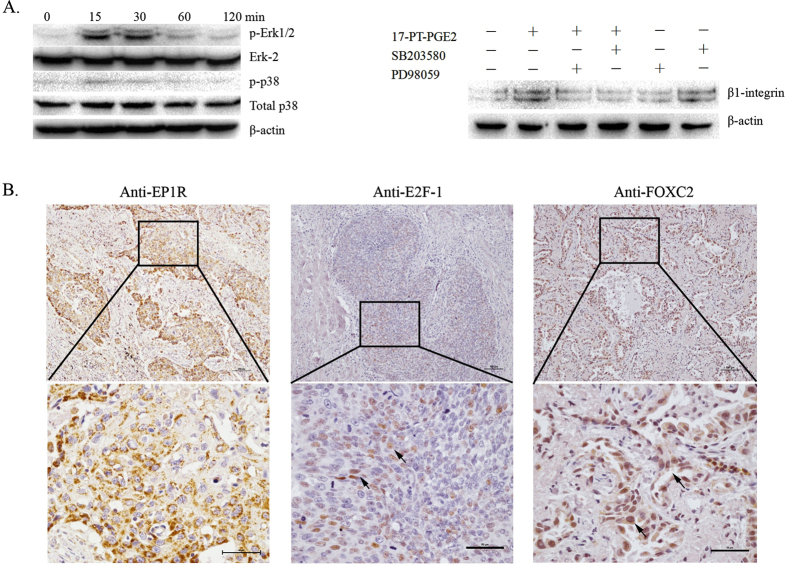
E2F-1 and FoxC2 are highly expressed in NSCLC tissues. (**A**) Effects of MAPKs on β1-integrin expression in NSCLC cells. A549 cells were treated with EP1a for various times. A549 cells were pre-treated with inhibitors of MEK (PD98059) or p38 (SB203580) for 1 h, followed by EP1a for 24 h. These experiments were performed three times with similar results. (**B**) The expression of E2F-1, FoxC2 and EP1R in lung cancer tissues. Representative immunohistochemical images of lung cancer tissues stained with anti-E2F-1, anti-FoxC2 and anti-EP1R antibodies. Scale bars = 100 μm (10×). Scale bars = 50 μm (40×).

E2F-1 functions in the downstream section of the PKC/MAPKs signalling pathway^21^,^22^. In the current study, the EP1 agonist improved both Erk1/2 and p38 phosphorylation in A549 cells. PD98059 and SB203580, which are MEK1/2 and p38 inhibitors, both suppressed EP1R-mediated β1-integrin upregulation. These data suggested that both Erk1/2 and p38 are involved in EP1R-mediated β1-integrin expression in NSCLC cells (Fig. 6A).

To observe the effects of E2F-1 and FoxC2 on COX-2-mediated β1-integrin expression in NSCLC tissues, sections were incubated with anti-EP1R, E2F-1 and FoxC2 antibodies. Using immunohistochemistry, we observed high expression of E2F-1, FoxC2 and EP1R (Fig. 6B). The positive signal for EP1R was mainly located in the cytoplasm, while the positive signal for E2F-1 was mainly located in the nuclei (arrow). FoxC2 was expressed in the cytoplasm and the nuclei (arrow).

## Discussion

COX-2-mediated production of PGE2 is involved in cell growth and metastasis in a number of cancers^23^,^24^,^25^,^26^. Our study showed that PGE2 treatment promoted cell migration and invasion in three NSCLC cell lines. However, the mechanism of PGE2-mediated cell growth and invasion in lung cancer is not clear. In NSCLC, increased expression of β1-integrin is associated with cell proliferation and migration^27^,^28^. Recently, β1 integrin was suggested as a prognostic biomarker for human lung adenocarcinoma^28^,^29^. However, little is known about the association between COX-2 and β1-integrin expression in lung cancer, and its related mechanisms. Our study suggested that COX-2 promoted cell invasion by increasing β1-integrin expression in NSCLC. COX-2 was over-expressed in NSCLC tissues. PGE2, the major product of COX-2, bound to EP1 receptor and activated PKC/MAPK signalling pathway; it went on to increase E2F-1 expression, which binding to the promoter of FoxC2 gene and promoting the expressions of FoxC2 and in turn, β1-integrin.

In our study, we used A549 and LLC cells in the experiments. We got two WB bands of β1-integrin in 130KD. We refered to the NCBI/Gene, and found that multiple alternatively spliced transcript variants which encode different protein isoforms have been found for this gene. COX-2 overexpression and exogenous PGE2 treatment caused cell migration and invasion, and β1-integrin upregulation in NSCLC cells. COX-2 siRNA downregulated β1-integrin expression *in vitro*. A lentivirus infection to silence β1-integrin expression led to diminished COX-2-promoted tumour growth and cell invasion, both *in vivo* and *in vitro*. Furthermore, COX-2 expression correlated with β1-integrin expression in NSCLC tissues. These data suggested that COX-2 promoted β1-integrin expression, and that β1-integrin was necessary for COX-2-mediated cell growth and invasion in NSCLC.

PGE2 exerts its effects by coupling to four subtypes of the EP receptor. Among the four receptor types, EP1R plays an important role in the development of many cancer cells^7^,^9^,^18^,^30^. Our studys showed that EP1R activation increased β1-integrin expression in NSCLC cells. The EP1 antagonist and EP1 siRNA decreased β1-integrin expression. Furthermore, EP1 silencing reduced tumour growth *in vivo*. EP1R mainly activates the PKC/MAPK signalling pathway to improve cell proliferation or invasion in various cancer cells^18^,^19^,^31^. In the current study, PKC also contributed to EP1R-mediated β1-integrin expression. These data suggested that EP1R is involved in tumour growth and β1-integrin expression in NSCLC. However, the mechanism of EP1R/PKC-mediated β1-integrin expression in lung cancer remains unclear. EP1 agonist treatment increased β1-integrin mRNA expression, which suggested that COX-2/EP1 modulated β1-integrin expression via transcriptional mechanisms.

FoxC2, a member of the family of winged helix/forkhead transcription factors, is reported to be involved in β1-integrin expression^20^. In the present study, FoxC2 siRNA suppressed β1-integrin expression and EP1R-mediated cell migration in NSCLC cells; the EP1 agonist improved FoxC2 expression; while Rottlerin suppressed EP1R-mediated FoxC2 expression significantly; ChIP assay identified that EP1 agonist treatment increased FoxC2 binding to β1-integrin promoter. MAPKs are involved in PKC downstream signalling pathway^32^,^33^. We found that the EP1 agonist improved both Erk1/2 and p38 phosphorylation in A549 cells, and that MEK1/2 and p38 inhibitors, suppressed EP1R-mediated β1-integrin upregulation. The involvement of the MAPK signalling pathway in EP1R-mediated β1-integrin expression suggested that some transcription factor(s) should bind to the FoxC2 promoter directly and be regulated by the Erk or p38 signalling pathways. Interestingly, there are two E2F-1-binding elements near the transcription initiation site of the FoxC2 gene.

E2F-1 is an important transcription factor involved in carcinogenesis and plays a major role in G1-S phase transition in various cancers^34^,^35^,^36^. MAPK-Erk and p38 are reported to modulate E2F-1 expression^22^,^37^. However, little was known about the effect of PGE2 on E2F-1 expression until now. The role of E2F-1 on β1-integrin expression is also unclear. Our study showed the both the EP1 agonist and PMA increased E2F-1 expression, and E2F1 siRNA blocked EP1R-mediated FoxC2 and β1-integrin upregulation. The ChIP and luciferase reporter assays revealed that EP1R activation improved FoxC2 transcription by the binding of E2F-1 to specific sequences in the promoter of FoxC2. These data suggested that E2F-1 plays an important role in COX-2-mediated β1-integrin expression and cell invasion in NSCLC cells.

In summary, our studies demonstrated that COX-2 increased β1-integrin expression in NSCLC, and that EP1 activation increased E2F-1 expression, by binding to the FoxC2 promoter and promoting the expressions of FoxC2 and in turn, β1-integrin. Our results increase our understanding of the mechanisms through which the COX-2/EP1R/MAPK/E2F-1 pathways regulate β1-integrin expression and cancer invasion, and may guide the future development of therapeutic interventions.

## Material and Method

### Materials

The NSCLC cell lines A549 and LLC were obtained from the American Type Culture Collection (ATCC, Manassas, VA, USA). The human NSCLC cell line H1299 was obtained from Jiangsu KeyGEN BioTECH Corporation (Nanjing, China). Dulbecco’s modified Eagle’s medium (DMEM) and Lipofectamine 2000 were from Invitrogen (Carlsbad, CA, USA). PGE_2_, 17-phenyl trinor-PGE_2_ (17-PT-PGE_2_), Butaprost, Sulprostone, PGE1 alcohol, sc51322, AH6809 and AH23848 were from Cayman Chemical Co (Ann Arbor, MI, USA). SB203580 (#559383), PD98059 (#513000), Rottlerin (#557370) and phorbol-12-myristate-13-acetate (PMA, #524400) were obtained from Merck (Darmstadt, Germany). The protein assay was from Bio-Rad (Hercules, CA, USA). Electrochemiluminescence (ECL) reagents were from Amersham Biosciences (Piscataway, NJ, USA). The transwell unit (12-well) was from Costar Corning Inc (Corning, NY, USA). Matrigel matrix was obtained from BD Bioscience (#356234, Bedford, MA, USA). G418 sulphate was from Amresco (Solon, OH, USA). The dual-luciferase reporter assay system was obtained from Promega Corporation (Madison, WI, USA). PrimeScript RT Reagent Kit was obtained from TAKARA Bio Inc. (#RR037A, Shiga, Japan). SYBGreen Master was obtained from Roche Diagnostics (#04913914001, Indianapolis, IN, USA). ChIP Assay Kit was obtained from Beyotime (#P2078, Shanghai, China). The following were commercially obtained antibodies: the anti-COX-2 antibody was obtained from Cayman Chemical Co. (Ann Arbor, MI, USA); the anti-human β1-integrin antibodies were obtained from BD Bioscience (#610467, Becton Dickinson, Franklin Lakes, NJ, USA) and Millipore Corporation (#AB1952P, Temecula, CA, USA); anti-mouse β1-integrin antibodies were obtained from R&D system (#MAB2405, Minneapolis, MN, USA); the anti-FoxC2 antibody was obtained from Abcam plc (#ab65141, Cambridge, UK); the anti-phosphorylated p38 antibody (#9215s) and anti-phosphorylated Erk1/2 antibody (#9106s) were obtained from Cell Signaling Technology (Danvers, MA, USA); the anti-p38α/β antibody (#sc-7972), anti-Erk2 antibody (#sc-154) and the anti-E2F-1 antibody (#sc-251) were obtained from Santa Cruz Biotechnology (Santa Cruz, CA, USA); the anti-β-actin antibody was obtained from Sigma Chemical Co. (St. Louis, MO, USA). EnVision + single reagents (Mouse, Rabbit) were from DAKO (K4000, K4002, Glostrup, Denmark).

## Methods

### Cell lines and culture

A549 cells, H1299 cells and LLC cells were cultured in DMEM with 10% foetal calf serum, 100 IU/ml penicillin and 100 g/ml streptomycin at 37 °C with 5% CO_2_.

### Animals

All animals (C57/B6 mice) were treated in accordance with the guidelines of the Institutional Animal Care and Use Committee at Nanjing Medical University. The normal diet was from Xietong Biotechnology Co. Ltd (Jiangsu, China). All animal experiments were carried out in accordance with the National Institutes of Health guide for the care and use of Laboratory animals (NIH Publications No. 8023, revised 1978). All experimental protocols were approved by the Institutional Animal Care and Use Committee at Nanjing Medical University, including any relevant details. All animal studies comply with the ARRIVE guidelines.

### Patients and specimens

Primary surgical specimens were obtained from 80 patients (aged from 52 to 83; average, 64) who were diagnosed clinically for squamous cell carcinoma or adenocarcinoma, from the Nanjing Chest Hospital and the Jiangsu Province Hospital. None of them had distant metastasis. All of them were approached for participation in the project. All experimental protocols were approved by the Human Ethics Committee of Nanjing Medical University, including any relevant details. The work conforms to the provisions of the Declaration of Helsinki in 1975. Written informed consent was obtained from all the donors for use of these samples in research. Resected specimens were fixed with neutral buffered 10% formalin and embedded in paraffin blocks. The diagnosis and histological grade of all the cases were confirmed independently by two pathologists, based on World Health Organization (WHO) classification.

### Immunohistochemical staining

Sections were treated with primary antibodies (anti-β1-integrin (#AB1952P, Millipore Corporation), COX-2, E2F-1 and FoxC2; 1:100 and 1:200 dilution) and incubated overnight at 4 °C. The specific signals were detected using EnVision polymer technology, and visualization was performed with 3,3N-diaminobenzidine tetrahydrochloride (DAB). The slices were photographed by under a LeiCa microscope and image analyse system. Four low power views (200×) were selected randomly from each sample in a blind manner; the level of integrated grey was estimated using the Q-Win software and presented as mean ± SEM.

### Cell migration assays

Cell migration assays were performed in 12-well transwell units. Pharmacological agents were added at the indicated times. After incubation at 37 °C for 12 h, the cells were fixed with ethanol and then stained with 0.1% crystal violet. The cells that had migrated to the lower surface of the membrane were solubilized with 10% acetic acid and quantified by measuring the absorbance at 590 nm.

### Cell invasion assays

Cell migration assays were performed in 12-well transwell units, coated with Matrigel Matrix. After incubation at 37 °C for 24 h, the cells were fixed and stained with crystal violet. The cells that had invaded to the lower surface of the membrane were solubilized with acetic acid and quantified by measuring the absorbance at 590 nm.

### Plasmid transfections

The pcDNA3-based plasmid encoding human COX-2 (COX2-pcDNA3) was a generous gift of Prof. Tong Wu in 2005 (School of Medicine, Tulane University, New Orleans, USA). The pcDNA3-based plasmid encoding the human EP1 receptor (EP1R-pcDNA3) was a generous gift of Dr. Kathy McCusker in 2007 (Merck Frosst Centre for Therapeutic Research, Canada). Lung cancer cells were transfected with the COX2-pcDNA3 plasmid, EP1R-pcDNA3 or pcDNA3 empty vector control using Lipofectamine 2000, according to manufacturer’s instructions. The efficiency of transfection was assayed by flow cytometry. The G418 antibiotic was used to select for cells stably expressing COX-2 or the EP1 receptor.

### siRNA transfection

The siRNA targeting the EP1 receptor (EP1R-siRNA) (ID: s194727) was obtained from Ambion. The siRNAs targeting COX-2 (COX-2 siRNA), E2F-1 (E2F1 siRNA1 and E2F1 siRNA2), and FoxC2 (FoxC2 siRNA) were obtained from Genepharma (Shanghai, China). The sequences were shown in Supplement 1.

A549 cells were transfected with the targeting siRNAs using Lipofectamine 2000. After 72 h, depletion of the target proteins was confirmed by Western blotting, and the cells were subsequently used for further experiments.

### Lentiviral infection

The silencing gene sequences targeting β1-integrin or EP1R were synthesized and cloned into the PLJM1 lentivirus vector (Addgene, Palo Alto, CA, USA). To produce the virus, pMD2.G and psPAX2 were co-transfected with PLJM1-silencing or the vehicle plasmid into 293T cells using Lipofectamine 2000 reagent. To obtain a stable EP1R or β1-integrin downregulated cell line, lentivirus-containing supernatant was harvested 48 h after transfection and used to infect LLC cells or COX-2- transfected LLC cells.

### Tumour xenografts models

Four-week-old female C57/B6 mice were injected with 5 × 10^6^/0.1 ml of various LLC cell lines into the flanks. Bi-dimensional tumour measurements were taken every two days. Tumour volume was measured along two major axes using callipers. Tumour volume (mm^3^) was calculated as follows: V = 1/2L × W^2^ (L: length, W: width). The mice were executed 21 days after injection; the tumour xenografts and adjacent lymph nodes were removed, and the specimens were fixed with neutral buffered 10% formalin and embedded in paraffin blocks. Sections (4 μM) of the tumour blocks were used for haematoxylin and eosin (HE) staining.

### Total RNA isolation and quantitative real-time PCR analysis

Total RNA was isolated from A549 cells using the Trizol reagent, according to the manufacturer’s instructions. For each sample, 0.5 μg of RNA was reverse transcribed using the PrimeScript RT Reagent Kit. Real time PCR analysis was performed using the SYBR Green PCR master mix. The sequences of the β1-integrin PCR primers and PCR reaction conditions were shown in Supplement 1.

### Western blotting

Cells were treated with pharmacological agents for various times, as indicated in the figure legends. The cells were collected and subjected to Western blotting analysis. The immunoreactivity was detected by using a standard enhanced-chemiluminescent reaction and analyzed using Image Lab 4.0 analysis software from Bio-Rad.

### ChIP-qPCR assay

ChIP-qPCR assays were performed using ChIP Assay Kit. Cells were crosslinked by addition of 1% formaldehyde at room temperature for 10 min. DNA was immunoprecipitated from the sonicated cell lysates by using antibodies against FoxC2, E2F-1 or IgG overnight at 4 °C and collected by incubation with protein A + G beads for 1 h. The complexes were eluted in elution buffer, treated with proteinase K. DNA was purified by qucick PCR purification Kit (Beyotime) and was used as a template for quantitative-PCR analysis. Fold enrichment was calculated based on Ct as 2−Δ(ΔCt), where ΔCt = CtIP − CtInput and Δ(ΔCt) = ΔCtantibody − ΔCtIgG. Primer sequences used in this study are listed in Supplement 1.

### Dual-Luciferase Reporter Assay

A549 cells were transiently transfected with a wild-type or truncated FoxC2 promoter-Luc reporter plasmid using Lipofectamine 2000 reagent. Cell lysates were prepared and luciferase activities were measured using the Promega Dual-Luciferase Reporter Assay system.

### Statistical analysis

Statistical analysis of the integrated grey level of β1-integrin and COX-2 was performed using STATA se12.0 software (StataCorp, Collage Station, TX, USA), and were analysed by Shapiro–Wilk’s W test, to detect whether the data distribution was normal or not and by Spearman’s correlation. Statistical significance was considered if P < 0.05.

Other data are presented as mean ± SEM. P-values were calculated by one-way ANOVA or Student’s t-test for unpaired samples using the GraphPad Prism software. The tests were two-tailed, the results were considered significant at P < 0.05.

## Additional Information

**How to cite this article**: Pan, J. *et al*. Cyclooxygenase-2 induced β1-integrin expression in NSCLC and promoted cell invasion via the EP1/MAPK/E2F-1/FoxC2 signal pathway. *Sci. Rep.* **6**, 33823; doi: 10.1038/srep33823 (2016).

## Supplementary Material

Supplementary Information

Supplementary Information 1

## Figures and Tables

**Table 1:** Clinicopathological features of squamous cell carcinoma patients and the levels of COX-2 and β1-integrin genes expression in NSCLC tissues.

		Number of patients	%	Molecular expression level			
Characteristic				COX-2	P-value	β1-integrin	P-value
All patients		40	100				
Grade	I–II	14	35	10743209 ± 560515	0.601	4289130 ± 252978	0.246
	III	26	65	11842314 ± 344665		3212700 ± 58658	
Lymph node	Y	21	53	12878544 ± 459750	0.372	3986202 ± 137292	0.403
Metastasis	N	19	47	10437197 ± 373175		3334740 ± 95137	
Stage	I–II	28	70	9721452 ± 246757	0.021*	3187400 ± 60588	0.049*
	III	12	30	16379625 ± 858472		4818592 ± 285638	

^*^P  < 0.05, Significant difference.

**Table 2:** Clinicopathological features of adenocarcinoma patients and the levels of COX-2 and β1-integrin genes expression in NSCLC tissues.

		Number of patients	%	Molecular expression level			
Characteristic				COX-2	P-value	β1-integrin	P-value
All patients		40	100				
Grade	I–II	20	50	6341278 ± 410556	0.378	2764136 ± 45456	0.501
	III	20	50	8295758 ± 267237		2954904 ± 43350	
Lymph node	Y	19	47	9890182 ± 428693	0.046*	3141653 ± 42670	0.097
Metastasis	N	21	53	4991774 ± 220203		2604256 ± 42107	
Stage	I–II	29	72	5326473 ± 142590	0.002**	2661041 ± 29399	0.019*
	III	11	28	12570272 ± 89291		3382782 ± 69493	

^*^P < 0.05, ^**^P < 0.01, Significant difference.
