# Supplementary material for: Cyclooxygenase-2 induced β1-integrin expression in NSCLC and promoted cell invasion via the EP1/MAPK/E2F-1/FoxC2 signal pathway
Source: Sci Rep. 2016 Sep 22;6:33823. doi: 10.1038/srep33823 (PMC5031967; doi:10.1038/srep33823)
Supplement: Supplementary Information [file srep33823-s1.pdf]

Cyclooxygenase-2 induced β1-integrin expression in NSCLC and promoted cell invasion via the EP1/MAPK/E2F-1/FoxC2 signal pathway

Jinshun Pan [a,b], Qinyi Yang [c], Jiaofang Shao [d], Li Zhang [b], Juan Ma [b], Yipin Wang [b], Bing-Hua Jiang [e], Jing Leng [b], Xiaoming Bai [b,*]

The siRNAs targeting following genes:

EP1R-siRNA: ACUUCUAAGCACAACCAGAtt (sense sequence).

COX-2 siRNA: CGGGAACACAACAGAGUAUtt (sense sequence).

E2F1 siRNA1: GACCACCUGAUGAAUAUCUtt (sense sequence).

E2F1 siRNA2: CUGCAGAGCAGAUGGUUAUtt (sense sequence).

FoxC2 siRNA: GACCCAACCAGACAAUUAAtt (sense sequence).

The sequences of the β1-integrin PCR primers used were:

Forward: 5'-CTGGAGATGGGAAACTTGGT-3'

Reverse: 5'-GTTTCTGGACAAGGTGAGCA-3'

PCR reaction conditions were: pre-incubation at 95°C for 10 min (1 cycle); 95°C for 15 s, 60°C for 15 s and 72°C for 30 s (40 cycles).

The sequences of the PCR primers used in β1-integrin promotor flanking FoxC2 binding elements were:

Forward: 5'- ATTCTACCCACCTAAGTTAACCAT -3'

Reverse: 5'- AGTATTGACTCCTTTTCAGCCCT -3'

The sequences of the PCR primers used in FoxC2 promotor flanking E2F-1 binding elements were:

Forward: 5'- GCTGCCAGGAGCCCG -3'

Reverse: 5'- CTGCATGCTGCTTCCGAGA-3'

PCR reaction conditions were: pre-incubation at 95°C for 10 min (1 cycle); 95°C for 15 s, 50°C for 15 s and 72°C for 30 s (40 cycles).
