# Supplementary material for: Cyclooxygenase-2 induced β1-integrin expression in NSCLC and promoted cell invasion via the EP1/MAPK/E2F-1/FoxC2 signal pathway
Source: Sci Rep. 2016 Sep 22;6:33823. doi: 10.1038/srep33823 (PMC5031967; doi:10.1038/srep33823)
Supplement: Supplementary Information 1 [file srep33823-s2.pdf]

Cyclooxygenase-2 induced β1-integrin expression in NSCLC and promoted cell invasion via the EP1/MAPK/E2F-1/FoxC2 signal pathway

Jinshun Pan [a,b], Qinyi Yang [c], Jiaofang Shao [d], Li Zhang [b], Juan Ma [b], Yipin Wang [b], Bing-Hua Jiang [e], Jing Leng [b], Xiaoming Bai [b,*]

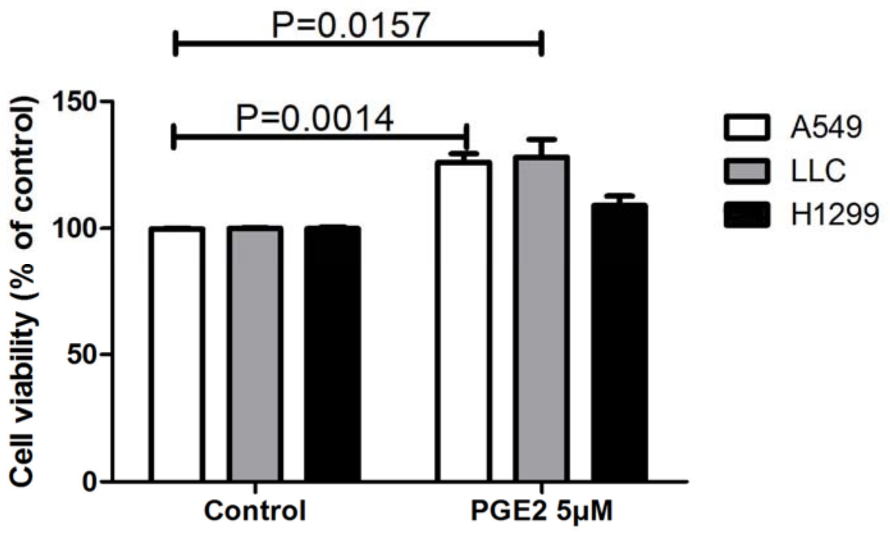

Figure S1. The effects of PGE2 on cell viabilities in NSCLC cells.
The cell viability assays were performed in 96-well units by CCK-8. A549, LLC and H1299 cells were treated with 5 μM PGE2. Results are presented as the mean ± SEM (n=3).
